# Association between dietary acid load and clinical features of migraine headaches among Iranian individuals

**DOI:** 10.1038/s41598-022-06515-x

**Published:** 2022-02-14

**Authors:** Keyhan Lotfi, Gholamreza Askari, Hamed Mohammad, Abdulmannan Fadel, Fariborz Khorvash, Arman Arab

**Affiliations:** 1 Department of Community Nutrition, School of Nutritional Sciences and Dietetics, Tehran University of Medical Sciences, Tehran, Iran; 2 Department of Community Nutrition, School of Nutrition and Food Science, Food Security Research Center, Isfahan University of Medical Sciences, Isfahan, Iran; 3 Department of Clinical Nutrition, School of Nutritional Sciences and Dietetics, Tehran University of Medical Sciences, Tehran, Iran; 4 School of Sport and Exercise Sciences, Faculty of Science, Liverpool John Moores University, Liverpool, UK; 5 Isfahan Neurosciences Research Center, Alzahra Hospital, Isfahan University of Medical Sciences, Isfahan, Iran

**Keywords:** Neurological disorders, Medical research

## Abstract

There is limited evidence regarding the possible role of dietary acid load (DAL) in the pathophysiology of migraine headaches. Therefore, we sought to examine DAL in relation to the clinical features of migraine including headache frequency, severity and duration, headache impact test-6 (HIT-6), and serum levels of nitric oxide (NO). In the present cross-sectional study, 262 patients (38 men and 224 women aged 20–50 years) were recruited through a simple random sampling method. Dietary intakes were obtained by using a validated 168-item semi-quantitative food frequency questionnaire (FFQ). DAL was then calculated by two different methods; potential renal acid load (PRAL) and net endogenous acid production (NEAP). In total, 262 patients with a mean (SE) age of 36.1 (0.53) and a BMI of 25.55 (0.21) were included in the current study. After controlling for potential confounders, a higher DAL was positively associated with headache frequency in those with the highest DAL score compared to the lowest (PRAL; β = 2.33; 95% CI 0.78, 3.88; NEAP; β = 1.74; 95% CI 0.13, 3.34). Increasing NEAP from 28.96 to 35.89 resulted in a 3.43 and 2.74 increment in HIT-6 scores in the crude (95% CI 1.35, 5.52) and fully-adjusted models (95% CI 0.40, 5.07), respectively. Moreover, a higher dietary PRAL was significantly associated with migraine-related disability, as shown by HIT-6, in subjects of the third tertile compared to those in the first tertile after controlling for confounders (β = 2.42; 95% CI 0.13, 4.70). In conclusion, our study highlighted the importance of the acid–base properties of a diet in the pathophysiology of migraine headaches. However, further well-designed studies are needed to confirm our findings.

## Introduction

Migraine is one of the most common neurological disorders worldwide that can cause substantial disability^1,2^. Globally, 18% and 6% of women and men, respectively are affected by migraine^3^. Recurrent episodes of headache and its concomitant symptoms (e.g., photophobia, phonophobia, nausea, and vomiting) which exist for 4–72 h are characterized as a migraine headache^4^. In addition to the substantial economic burden on societies that are imposed by migraine^5^, this condition has been linked to several health problems such as impaired cognition chronic heart disease, stroke, obesity, depression, and anxiety^6,7^.

Appropriate acid–base balance is a key factor for the normal physiological function of the body^8^. An imbalance in acid–base homeostasis can be linked with various diseases, such as diabetes, cardiovascular disease, Alzheimer’s disease, and mood disorders^8^. Chronic metabolic acidosis could also invoke hypertension, which is a potential precursor of migraine headaches^9,10^. Furthermore, a positive relation between acidosis and nitric oxide (NO) synthesis has been found^11^, NO has been suggested as a possible mechanism by which migraine attacks might be aggravated^12^.

Maintaining acid–base equilibrium by consuming beneficial foods has gained interest in recent years^13^. This could prevent metabolic acidosis which deleteriously affects human health^13^. Diets characterized by high intakes of meat, dairy products, and grains, as well as low intake of fruit and vegetables can lead to metabolic acidosis^14^. Dietary acid load (DAL) has been established as a means of detecting the balance between acidic foods (e.g. animal sources of protein) and alkaline foods (e.g. vegetables and fruit) and to provide the acid–base properties of the diet^14^.

The association between specific food groups and migraine has been investigated previously^15,16^. However, studies on the association between DAL and migraine have not been widely performed. A cross-sectional study found a higher DAL to be deleteriously associated with migraine headache characteristics among women^17^. Since previous studies focused mostly on specific foods and found an inverse association between alkaline foods and migraine headache^15^, there may be also a link between DAL and migraine. The association between DAL and migraine headache was previously investigated in women, and thus such association remains unsolved among men. Therefore, this study was carried out to investigate the association between DAL and clinical outcomes of migraine headaches including severity, frequency, and duration in a sample of Iranian adults.

## Materials and methods

### Participants

The current cross-sectional study was performed among Iranian adults between August 2019 and June 2020. Using a simple random sampling method, a total sample of 262 patients aged from 20 to 50 years was recruited from neurology clinics affiliated with Isfahan University of Medical Sciences. Patients were eligible for the present study if they: (1) had a body mass index (BMI) between 18.5 and 30 kg/m^2^ and (2) were diagnosed with migraine by an expert neurologist based on the International Classification of Headache Disorders (ICHD–3) criteria^18^. Those who had a history of diabetes, cardiovascular disease, hypertension, cancer, hepatic, thyroid, or renal disease, and other neurological disorders, as well as those who had used dietary and herbal supplements (i.e. magnesium, coenzyme Q10, riboflavin, or feverfew) were excluded. Also, individuals who reported daily energy intakes outside the range of 800–4200 kcal/day (3347–17,573 kJ/day) were excluded from the current analysis^19^. This study was approved by the Isfahan University of Medical Sciences Research Ethics Committee (IR.MUI.RESEARCH.REC.1398.352). All participants provided written informed consent forms. All study protocols were conducted according to the ethical guidelines of the 1975 Declaration of Helsinki.

### Dietary intake assessment

We applied an interviewer-administered 168-item food frequency questionnaire (FFQ) to gather individuals’ usual dietary intakes during the previous year. Its validity and reliability have been reported previously^20^. Foods with their standard serving sizes were listed in this FFQ with a daily, weekly, or monthly frequency basis option. A trained dietitian asked the participants about the consumption frequency for each food item over the preceding year. Considering the reported portion size and frequency for each food, all food items were calculated on a daily basis and then were converted to grams per day using household measures^21^. Energy and the other nutrients’ content for each item were then computed using Nutritionist 4 software (First Databank, Hearst Corp., San Bruno, CA, USA), which had been modified for Iranian foods.

### Calculating DAL

Potential renal acid load (PRAL) and net endogenous acid production (NEAP) are two different validated^22,23^ methods that were used to calculate DAL. They were computed using the algorithms below:

- PRAL (mEq/day) = (protein intake [g/day] × 0.49) + (phosphorus intake [mg/day] × 0.037) − (potassium intake [mg/day] × 0.021) − (calcium intake [mg/day] × 0.013) − (magnesium intake [mg/day] × 0.026).
- NEAP (mEq/day) = (54.5 × protein intake [g/day] ÷ potassium intake [mEq/day]) − 10.2.

Higher values show that the diet could potentially shift the acid–base equilibrium into acidosis.

### Assessment of migraine clinical outcomes

The possible impact of migraine on participants’ quality of life was examined by a 6-item validated questionnaire, headache impact test (HIT-6)^24^. This questionnaire contains a five-level option for each question: never (6 scores), rarely (8 scores), sometimes (10 scores), very often (11 scores), and always (13 scores). The overall score ranges were from 36 to 78. The effect of headache on the individuals’ quality of life was categorized to be none (36–49), moderate (50–55), substantial (56–59), and severe (≥ 60).

Participants were asked to fill out a 30-day headache diary which was accompanied by written and verbal instructions throughout the upcoming month. In this diary, clinical features of migraine headache including time of attack onset, duration, and severity had to be reported. The visual analog scale (VAS) questionnaire was applied to assess the severity of headaches^25^. Each participant received a score ranging from 0 to 10, in which “0” meant no pain and “10” the worst imaginable pain. The number of attacks per month (frequency) and mean duration of headache attacks per month (duration) were also examined.

### Assessment of serum NO

Following 8–12 h of fasting, 5 mL of venous blood was taken from participants in the Baradaran laboratory, affiliated with Isfahan University of Medical Sciences. The blood samples were centrifuged at 3500 rpm and serum was separated and kept at − 80 °C for later analysis. Serum NO was measured using available commercial kits (Kiazist Life Sciences, Iran) using the Griess method.

### Assessment of other variables

Data on demographic characteristics including gender, age, smoking, marital status, number of family members, and medications were gathered through a face-to-face interview. We used an Iranian validated version of the International Physical Activity Questionnaire (IPAQ)^26^ for 7 days to examine individuals’ physical activity status. To measure blood pressure, a mercury sphygmomanometer (Riester, Germany) was applied. Weight was measured using a digital scale (Omron BF511, Omron Corp., Kyoto, Japan) in light clothes to the nearest 100 g. Height was measured to the nearest 1 mm by a wall tape meter in a standing position without shoes. The BMI was then calculated using weight (kg) divided by height squared (m^2^).

### Statistical analysis

After constructing PRAL and NEAP, participants were classified based on tertiles of both PRAL and NEAP. Continuous and categorical variables were expressed as mean ± standard error (SE) and number (percent), respectively. To compare differences in qualitative and quantitative variables across tertiles of PRAL and NEAP, the Chi-square test and analysis of variance (ANOVA) were applied, respectively. Multiple linear regression analysis was used to evaluate the association between DAL (PRAL and NEAP) and headache frequency, duration, severity, HIT-6, and serum NO levels in crude and adjusted models. The first model was adjusted for age (continuous), sex, and energy intake (continuous). The second model was further controlled for smoking status (current smoker/non-current smoker), marital status (single/married), migraine characteristic (with aura/without aura), family history (yes/no), physical activity (continuous), and mean arterial pressure (continuous). A third model included those variables in Model 2 plus BMI. All beta (β) estimates were obtained by considering the first tertile of PRAL and NEAP as the reference. The tertiles of PRAL and NEAP were considered as ordinal variables in the linear regression models to estimate the trend of β across these tertiles. All the analyses were performed by applying SPSS version 26 (IBM Corp, Armonk, NY, USA). P-values < 0.05 were considered significant.

### Ethics approval and consent to participate

The research ethics committee of Isfahan University of Medical Sciences approved the protocol of the current study on 26 August 2019 (IR.MUI.RESEARCH.REC.1398.352).

## Results

In total, 262 patients with a mean (SE) age of 36.1 (0.53) and a BMI of 25.55 (0.21) were included in the current study. Characteristics of study participants across the tertiles of dietary PRAL and NEAP are indicated in Table 1. Subjects in the last tertile of PRAL were less likely to be female (P = 0.018) and had lower age (P = 0.012) as well as higher headache frequency (P < 0.001), compared to the first tertile. Furthermore, individuals in the top versus bottom tertiles of NEAP had a lower age (P = 0.022) and earlier diagnosis of migraine (P = 0.027) as well as a higher headache frequency (P = 0.001) and HIT-6 score (P = 0.03). There was no significant difference in the case of other variables provided in Table 1 (all P-values > 0.05).

**Table 1:** Characteristics of study population stratified by tertiles of dietary PRAL and NEAP.

Variables	Teriles of dietary PRAL				Teriles of dietary NEAP			
	T1 [< − 22.50 (mEq/day)]	T2 [− 22.50 to − 11.06 (mEq/day)]	T3 [> − 11.06 (mEq/day)]	P value	T1 [< 28.96 (mEq/day)]	T2 [28.96 to 35.89 (mEq/day)]	T3 [> 35.89 (mEq/day)]	P value
N	87	88	87		87	88	87	
PRAL (mEq/day)	− 30.86 ± 1.50	− 16.96 ± 0.36	− 4.06 ± 1.46	< 0.001	− 30.11 ± 1.55	− 17.02 ± 0.70	− 4.75 ± 1.43	< 0.001
NEAP (mEq/day)	26.49 ± 0.80	32.72 ± 0.49	42.93 ± 1.42	< 0.001	25.69 ± 0.79	32.48 ± 0.20	43.97 ± 1.36	< 0.001
**Demographic variables**								
Age (years)	37.01 ± 0.89	37.40 ± 0.88	33.87 ± 0.95	0.012	37.56 ± 0.85	36.64 ± 0.91	34.09 ± 0.96	0.022
Female	79 (90.8)	77 (87.5)	68 (78.2)	0.018	76 (87.4)	77 (87.5)	71 (81.6)	0.283
Married	70 (80.5)	75 (85.2)	67 (77.0)	0.564	74 (85.1)	71 (80.7)	67 (77.0)	0.178
Current smoker	2 (2.3)	7 (8.0)	6 (6.9)	0.193	3 (3.4)	6 (6.8)	6 (6.9)	0.329
Number of family members	3.47 ± 0.10	3.32 ± 0.11	3.44 ± 0.10	0.598	3.39 ± 0.10	3.53 ± 0.97	3.32 ± 0.11	0.352
Weight (kg)	67.59 ± 1.03	68.73 ± 1.08	67.13 ± 1.31	0.601	68.81 ± 1.06	67.24 ± 1.08	67.41 ± 1.29	0.571
Height (cm)	162.17 ± 0.80	162.44 ± 0.90	163.94 ± 0.79	0.273	162.08 ± 0.89	162.57 ± 0.81	163.90 ± 0.78	0.281
BMI (kg/m^2^)	25.72 ± 0.36	26.03 ± 0.34	24.88 ± 0.38	0.072	26.22 ± 0.35	25.42 ± 0.34	25.00 ± 0.38	0.059
Physical activity (MET/h/day)	10.27 ± 2.87	6.73 ± 1.49	9.65 ± 2.08	0.485	9.96 ± 2.86	7.67 ± 1.61	9.01 ± 2.00	0.763
SBP (mmHg)	112.24 ± 1.14	112.23 ± 1.05	113.50 ± 0.91	0.613	113.44 ± 1.07	111.98 ± 1.05	112.55 ± 0.99	0.608
DBP (mmHg)	75.66 ± 0.68	74.56 ± 0.87	76.22 ± 0.75	0.306	76.68 ± 0.59	74.73 ± 0.79	75.03 ± 0.89	0.160
MAP (mmHg)	87.85 ± 0.78	87.12 ± 0.84	88.65 ± 0.72	0.391	88.94 ± 0.70	87.15 ± 0.79	87.54 ± 0.85	0.242
**Migraine-related information**								
Migraine in first degree relatives	59 (67.8)	55 (62.5)	53 (60.9)	0.345	53 (60.9)	61 (69.3)	53 (60.9)	> 0.99
Time since migraine diagnosis (year)	7.63 ± 0.92	8.44 ± 0.99	5.91 ± 0.85	0.145	8.88 ± 1.02	7.70 ± 0.83	5.40 ± 0.90	0.027
Episodic migraine	74 (85.1)	70 (79.5)	72 (82.8)	0.691	71 (81.6)	74 (84.1)	71 (81.6)	> 0.99
Migraine with aura	41 (47.1)	38 (43.2)	30 (34.5)	0.091	41 (47.1)	37 (42.0)	31 (35.6)	0.125
Frequency (attacks per month)	5.60 ± 0.45	7.97 ± 0.73	9.82 ± 0.92	< 0.001	6.35 ± 0.48	7.03 ± 0.68	10.03 ± 0.95	0.001
Duration (day/attack)	1.03 ± 0.09	0.99 ± 0.09	0.87 ± 0.08	0.432	0.90 ± 0.08	1.12 ± 0.10	0.86 ± 0.07	0.089
Severity (visual analogue scale)	7.89 ± 0.20	7.95 ± 0.17	7.48 ± 0.19	0.163	7.89 ± 0.20	7.88 ± 0.17	7.55 ± 0.19	0.349
HIT-6	61.50 ± 0.81	62.86 ± 0.70	63.79 ± 0.76	0.107	61.23 ± 0.78	62.84 ± 0.74	64.09 ± 0.74	0.030
Nitric oxide (nmol/mL)	35.37 ± 2.30	32.85 ± 2.26	34.17 ± 2.28	0.738	32.81 ± 2.15	36.79 ± 2.56	32.74 ± 2.08	0.355
**Medications**								
Taking beta-blockers	33 (37.9)	36 (40.9)	39 (44.8)	0.356	36 (41.4)	30 (34.1)	42 (48.3)	0.356
Taking topitamate	5 (5.7)	3 (3.4)	5 (5.7)	> 0.99	4 (4.6)	4 (4.5)	5 (5.7)	0.728
Taking TCAs	39 (44.8)	46 (52.3)	37 (42.5)	0.762	40 (46.0)	41 (46.6)	41 (47.1)	0.879
Taking TeCAs	3 (3.4)	3 (3.4)	2 (2.3)	0.660	2 (2.3)	3 (3.4)	3 (3.4)	0.660
Taking SNRIs	2 (2.3)	5 (5.7)	7 (8.0)	0.093	3 (3.4)	6 (6.8)	5 (5.7)	0.501
Taking sodium valproate	11 (12.6)	8 (9.1)	14 (16.1)	0.494	11 (12.6)	11 (12.5)	11 (12.6)	> 0.99
Taking triptans	15 (17.2)	15 (17.0)	13 (14.9)	0.683	15 (17.2)	14 (15.9)	14 (16.1)	0.838
Taking gabapentin	17 (19.5)	14 (15.9)	12 (13.8)	0.307	17 (19.5)	12 (13.6)	14 (16.1)	0.540
Taking benzodiazepine	1 (1.1)	7 (8.0)	5 (5.7)	0.163	2 (2.3)	7 (8.0)	4 (4.6)	0.486

Data are presented as mean ± standard error or number (% within tertiles of dietary PRAL and NEAP).

*BMI* body mass index, *SBP* systolic blood pressure, *DBP* diastolic blood pressure, *MAP* mean arterial pressure, *TCA* tricyclic antidepressants, *TeCA* tetracyclic antidepressant, *SNRI* serotonin-norepinephrine reuptake inhibitor, *HIT* headache impact test, *PRAL* potential renal acid load, *NEAP* net endogenous acid production.

P-value obtained from chi-square analysis for categorical variables and analysis of variance (ANOVA) for continuous variables.

Dietary intakes of selected nutrients and food groups across tertiles of PRAL and NEAP are presented in Table 2. Compared to the lowest tertile, participants in the highest tertile of both PRAL and NEAP had significantly higher intakes of protein, fat, and meat as well as lower intakes of carbohydrate, potassium, magnesium, fruit, and vegetables. Furthermore, participants in the top tertile of PRAL had a significantly higher energy intake compared to the bottom tertile. In contrast; considering the highest versus lowest tertile of NEAP, subjects consumed less energy.

**Table 2:** Selected food groups and nutrients intake of participants across tertiles of dietary PRAL and NEAP.

Variables	Tertiles of dietary PRAL				Tertiles of dietary NEAP			
	T1	T2	T3	P-value	T1	T2	T3	P-value
**Nutrients**								
Energy (kcal/day)	2729.63 ± 71.52	2472.21 ± 67.16	2741.10 ± 72.59	0.012	2772.56 ± 72.24	2459.21 ± 60.82	2735.52 ± 77.93	0.003
Protein (g/day)	71.05 ± 1.53	70.87 ± 1.67	77.68 ± 2.56	0.023	67.37 ± 1.51	72.90 ± 1.58	80.18 ± 2.65	< 0.001
Fat (g/day)	105.89 ± 2.15	111.93 ± 2.07	114.99 ± 2.37	0.012	109.14 ± 2.38	108.97 ± 1.90	115.02 ± 2.41	0.105
Carbohydrate (g/day)	379.68 ± 4.55	361.12 ± 4.48	343.18 ± 5.38	< 0.001	374.24 ± 4.99	365.22 ± 4.25	342.78 ± 5.56	< 0.001
Potassium (mg/day)	4331.55 ± 83.67	3556.25 ± 72.46	3119.19 ± 94.66	< 0.001	4196.99 ± 94.76	3633.76 ± 81.10	3136.83 ± 95.67	< 0.001
Magnesium (mg/day)	301.29 ± 5.67	274.55 ± 5.76	258.73 ± 6.82	< 0.001	291.94 ± 6.42	282.75 ± 5.70	258.26 ± 6.68	0.001
Calcium (mg/day)	1059.84 ± 43.28	1001.06 ± 41.86	1013.86 ± 53.78	0.646	1016.81 ± 40.98	1056.17 ± 51.39	1000.86 ± 47.29	0.691
Phosphorous (mg/day)	1179.17 ± 33.70	1168.88 ± 34.87	1233.31 ± 42.11	0.421	1156.50 ± 35.39	1221.20 ± 38.42	1206.03 ± 37.51	0.428
**Food groups (g/day)**								
Fruit	755.04 ± 32.46	465.90 ± 23.18	385.73 ± 22.83	< 0.001	739.98 ± 34.67	460.46 ± 22.39	400.47 ± 23.64	< 0.001
Vegetables	428.45 ± 26.13	266.49 ± 16.19	233.50 ± 15.22	< 0.001	412.52 ± 25.92	273.40 ± 16.27	240.08 ± 17.96	< 0.001
Meat	29.54 ± 3.11	30.00 ± 3.74	49.53 ± 5.82	0.001	27.37 ± 2.95	29.66 ± 3.04	54.37 ± 6.54	< 0.001
Fish	4.29 ± 0.75	4.09 ± 0.72	4.66 ± 0.55	0.834	4.12 ± 0.75	4.16 ± 0.65	4.84 ± 0.61	0.715
Whole grains	46.28 ± 6.03	38.08 ± 4.02	35.41 ± 4.61	0.273	44.46 ± 5.68	36.80 ± 4.21	38.62 ± 5.09	0.522

Data are presented as mean ± standard error and obtained from analysis of variance (ANOVA).

*PRAL* potential renal acid load, *NEAP* net endogenous acid production.

P < 0.05 was considered statistically significant.

The β estimates and 95% CIs for headache frequency, duration, severity, HIT-6, and serum NO are shown in Table 3. In the crude model, subjects in the third tertile of PRAL had a higher headache frequency (β = 4.21; 95% CI 2.20, 6.23), compared to the first tertile. A similar result was found for the association between NEAP and headache frequency (β = 3.67; 95% CI 1.65, 5.70). After adjustment for age, sex and energy intake, the associations were attenuated for PRAL (β = 3.35; 95% CI 1.25, 5.45) and NEAP (β = 2.41; 95% CI 0.20, 4.62). The results were further attenuated for both PRAL (β = 2.31; 95% CI 0.75, 3.86) and NEAP (β = 1.71; 95% CI 0.11, 3.32) after additional adjustment for marital status, smoking status, migraine type, migraine characteristics, family history, mean arterial pressure, and physical activity. After controlling for BMI, a higher DAL was positively associated with headache frequency in those with the highest DAL score compared to the lowest (PRAL; β = 2.33; 95% CI 0.78, 3.88; NEAP; β = 1.74; 95% CI 0.13, 3.34). A higher dietary PRAL was significantly associated with migraine-related disability, as shown by HIT-6, in subjects of the third tertile compared to those in the first tertile, either in the crude model (β = 2.28; 95% CI 0.17, 4.39) or after controlling for age, sex and energy intake (β = 2.42; 95% CI 0.13, 4.70). Further adjustment for marital status, smoking status, migraine type, migraine characteristic, family history, mean arterial pressure and physical activity attenuated the findings (β = 2.11; 95% CI − 0.18, 4.41). Also, in the fully adjusted model, the association between PRAL and HIT-6 was not statistically significant (β = 2.04; 95% CI − 0.24, 4.33). Conversely, increasing NEAP from 28.96 to 35.89 resulted in a 3.43 and 2.74 increment in HIT-6 scores in the crude (95% CI 1.35, 5.52) and fully-adjusted models (95% CI 0.40, 5.07), respectively. In addition, both PRAL and NEAP were not found to be significantly associated with headache duration, headache severity, and serum NO either before or after adjustment for potential confounders.

**Table 3:** Beta (β) and 95% confidence interval for clinical features of migraine headache according to tertiles of dietary PRAL and NEAP.

	Tertiles of dietary PRAL				Tertiles of dietary NEAP			
	T1	T2	T3	P trend	T1	T2	T3	P trend
**Frequency**								
Crude	Ref	2.36 (0.36, 4.37)	4.21 (2.20, 6.23)	< 0.001	Ref	0.67 (− 1.33, 2.69)	3.67 (1.65, 5.70)	< 0.001
Model 1	Ref	2.65 (0.52, 4.77)	3.35 (1.25, 5.45)	0.002	Ref	0.66 (− 1.45, 2.78)	2.41 (0.20, 4.62)	0.035
Model 2	Ref	1.24 (− 0.32, 2.81)	2.31 (0.75, 3.86)	0.004	Ref	− 0.04 (− 1.59, 1.50)	1.71 (0.11, 3.32)	0.042
Model 3	Ref	1.19 (− 0.37, 2.76)	2.33 (0.78, 3.88)	0.003	Ref	− 0.01 (− 1.56, 1.52)	1.74 (0.13, 3.34)	0.038
**Duration**								
Crude	Ref	− 0.03 (− 0.28, 0.20)	− 0.15 (− 0.40, 0.08)	0.212	Ref	0.21 (− 0.02, 0.46)	− 0.03 (− 0.27, 0.20)	0.777
Model 1	Ref	− 0.05 (− 0.33, 0.21)	− 0.06 (− 0.33, 0.21)	0.662	Ref	0.22 (− 0.04, 0.49)	0.05 (− 0.22, 0.33)	0.638
Model 2	Ref	− 0.05 (− 0.33, 0.22)	− 0.08 (− 0.35, 0.19)	0.563	Ref	0.21 (− 0.05, 0.48)	0.03 (− 0.24, 0.31)	0.751
Model 3	Ref	− 0.04 (− 0.32, 0.22)	− 0.08 (− 0.35, 0.18)	0.544	Ref	0.21 (− 0.05, 0.48)	0.03 (− 0.24, 0.30)	0.775
**Severity**								
Crude	Ref	0.05 (− 0.46, 0.58)	− 0.41 (− 0.93, 0.11)	0.123	Ref	− 0.01 (− 0.53, 0.51)	− 0.34 (− 0.87, 0.18)	0.200
Model 1	Ref	0.14 (− 0.41, 0.71)	0.04 (− 0.50, 0.60)	0.861	Ref	0.08 (− 0.47, 0.63)	0.02 (− 0.55, 0.60)	0.926
Model 2	Ref	0.08 (− 0.47, 0.64)	− 0.07 (− 0.63, 0.48)	0.802	Ref	0.11 (− 0.44, 0.66)	0.006 (− 0.56, 0.57)	0.968
Model 3	Ref	0.07 (− 0.48, 0.64)	− 0.06 (− 0.62, 0.48)	0.816	Ref	0.11 (− 0.43, 0.66)	0.01 (− 0.56, 0.58)	0.951
**HIT-6**								
Crude	Ref	1.35 (− 0.74, 3.46)	2.28 (0.17, 4.39)	0.034	Ref	1.89 (− 0.18, 3.97)	3.43 (1.35, 5.52)	0.001
Model 1	Ref	1.37 (− 0.94, 3.68)	2.42 (0.13, 4.70)	0.038	Ref	1.18 (− 1.08, 3.44)	3.06 (0.69, 5.42)	0.012
Model 2	Ref	0.87 (− 1.44, 3.19)	2.11 (− 0.18, 4.41)	0.071	Ref	0.73 (− 1.52, 2.99)	2.82 (0.48, 5.17)	0.020
Model 3	Ref	1.01 (− 1.29, 3.32)	2.04 (− 0.24, 4.33)	0.080	Ref	0.64 (− 1.60, 2.90)	2.74 (0.40, 5.07)	0.023
**Nitric oxide**								
Crude	Ref	− 2.51 (− 8.80, 3.77)	− 1.19 (− 7.50, 5.10)	0.710	Ref	3.96 (− 2.30, 10.24)	− 0.09 (− 6.38, 6.19)	0.977
Model 1	Ref	− 5.09 (− 12.10, 1.92)	− 2.10 (− 9.03, 4.83)	0.535	Ref	3.77 (− 3.14, 10.69)	1.28 (− 5.94, 8.51)	0.693
Model 2	Ref	− 3.19 (− 10.20, 3.81)	− 0.43 (− 7.38, 6.50)	0.893	Ref	4.69 (− 2.16, 11.54)	2.35 (− 4.75, 9.46)	0.486
Model 3	Ref	− 2.99 (− 10.01, 4.02)	− 0.54 (− 7.48, 6.39)	0.868	Ref	4.55 (− 2.29, 11.41)	2.23 (− 4.87, 9.33)	0.509

Data are presented as β (95% confidence interval) and obtained from linear regression.

Crude: Unadjusted.

Model 1: Adjusted for age, sex, and energy intake.

Model 2: Model 1 + marital status, smoking status, migraine type, migraine characteristic, family history, mean arterial pressure, and physical activity.

Model 3: Model 2 + body mass index.

*HIT* headache impact test, *PRAL* potential renal acid load, *NEAP* net endogenous acid production.

P < 0.05 was considered statistically significant.

## Discussion

The present study is among the first to address the potential roles of DAL in migraine headaches using a sample of Iranian individuals diagnosed with migraine. We found that dietary PRAL and NEAP are independent predictors of headache frequency. Moreover, there was a significant association between NEAP and PRAL and migraine-related disability; however, this link was dependent on potential confounders for PRAL. Our findings highlighted the importance of acid–base properties of a diet in the pathophysiology of migraine headaches and contribute to the current literature to provide new information regarding the role of diet in migraine headaches in a sample of migraine patients seeking care from specialty clinics.

Migraine is considered the first cause of disability in adults under 50, and its growing prevalence can impose a detrimental effect on public health^27^. Migraine can also lead to other chronic diseases and mortality^28,29^, and needs special attention. We found a positive association between the acidity of the diet and some migraine headache features. Therefore, it is clinically important to recommend migraine patients to consume alkaline foods such as vegetables, fruits, nuts, and legumes as well as limiting acidifying foods including meats and meat products. This approach could be done in the context of plant-based diets such as the Mediterranean diet^30^ or the dietary approaches to stop hypertension (DASH) diet^31^.

We found that the association between PRAL and HIT-6 was not independent of marital status, smoking status, migraine type, migraine characteristics, family history, mean arterial pressure, physical activity, and BMI, indicating that the potential effect of PRAL is related to various lifestyle and clinical aspects of migraine patients and that it is not specific to PRAL alone. Previously, a direct association between obesity and migraine characteristics was shown^32–34^. An inverse association between adiposity and health-related quality of life was also investigated^35^. Relations between migraine headache and smoking^36,37^, physical activity^38^, family history of migraine^39^, and blood pressure^40^ was found in the prior studies. Also, lower quality of life could be associated with smoking habits^41^, less physical activity^42–44^, and having hypertension^45^. On the other hand, PRAL has been shown to be related to hypertension^46^ and obesity^47^. Studies have suggested that individuals with higher physical activity levels have a higher consumption of fruit and vegetables, which have a less acidifying effect^48,49^. Given the potential mediating effect of the above-mentioned confounders, the insignificant association between PRAL and HIT-6 in the adjusted models might be explained.

In the current study, PRAL and NEAP were independently used to calculate DAL. We found that individuals in the highest tertile of PRAL and NEAP have higher intakes of protein and lower intakes of potassium and magnesium. We also showed that patients in the highest tertile of PRAL and NEAP have higher intakes of meat and lower intakes of fruit and vegetables. Higher intakes of protein and phosphorus contribute to higher scores of PRAL and NEAP, indicating potential acidity of the diet^50^, whereas higher intakes of potassium, magnesium, and calcium result in lower scores of PRAL and NEAP^50^. It is worth mentioning that the quality of protein is also an important factor. Animal proteins have higher contents of phosphorus that increase PRAL and, therefore, have an acidifying effect on the diet^13^. Furthermore, hydrochloric acid is generated in the metabolism of arginine, lysine, and histidine, which are highly prevalent in animal proteins^14^. Studies have suggested that milk and dairy products, as animal sources of protein, are rich in calcium that compensates for their high phosphorous content^13^. In contrast to animal proteins, phosphorous found mostly in the form of phytate in vegetable proteins has less bioavailability^13^. Also, vegetable proteins are high in glutamate which requires hydrogen ions for their metabolism, and thus, vegetable proteins have a neutral effect on the acid load^51^. In addition, fruit and vegetables are generally rich in potassium and magnesium that resulting in a higher alkalinizing capacity of the diet^13^. Given the above-mentioned points, animal-based foods such as meat and meat products are responsible for the potential acidity of the diet, and fruit and vegetables act as protective determinants for metabolic acidosis. Therefore, a diet high in fruit and vegetables, with reduced meat intake, could be recommended to maintain the acid–base equilibrium in the body.

In the previous studies, specific foods have been investigated mostly in relation to migraine headaches. A case–control study found that those who have migraine headaches have higher intakes of red and white meat, whereas they consume lower amounts of fruit and vegetables compared to healthy controls^52^. Furthermore, another case–control study found fruit and vegetables to be inversely associated with the odds of migraine among children^15^. Conversely, a cross-sectional study on 11,910 Canadian adults did not observe a significant relationship between fruit and vegetables intake and risk of migraine^53^. Another cross-sectional study revealed that a dietary pattern high in meat and meat products, known as the Western dietary pattern, is inversely related to the odds of migraine^54^. However, no significant association was depicted between the dietary pattern rich in fruit and vegetables and migraine risk^54^. Nevertheless, we must keep in mind that humans consume a combination of different nutrients and foods; therefore, it is more important to take the whole diet into account when examining the acidifying or alkalinizing effects of foods concerning diseases.

DAL has not been widely investigated in relation to migraine headaches. In opposition to our findings, a cross-sectional study found that women in the top tertile of PRAL and NEAP were more likely to experience severe headaches compared to the bottom tertile^17^. It was also found that PRAL and NEAP scores were directly correlated to the duration of headaches^17^. A case–control study revealed that adults in the highest tertile of PRAL and NEAP have a seven and fourfold increase in the odds of having migraines^55^. These discrepancies might be explained by different study designs, sample sizes, and methods for assessing dietary intake and the outcome. Also, considering different confounders in the studies might result in inconsistent results.

An essential concern in epidemiologic studies, especially in cross-sectional studies, is the possible existence of reverse causation. The reverse causation hypothesis is that the relation might be in the reverse direction, for which the study is conducted^56^. It is worth noting that migraine patients might change their dietary habits, and thus, a bidirectional association might exist between DAL and migraine headaches. Given the lack of studies examining such associations, further prospective cohort studies on large populations are essential to elucidate the causal relationship between DAL and migraine headaches.

The association between DAL and migraine headaches could be explained through several mechanisms. Acidosis has been suggested to augment NO and inflammatory marker production^11,57,58^. Consequently, migraine headaches can be initiated by the action of the NO-cyclic guanosine monophosphate (cGMP) pathway^12^. Also, NO and TNF-α can prolong the pain by stimulating calcitonin gene-related peptide release, a potential factor triggering migraine headaches, in trigeminal ganglion neurons^12^. However, we did not find a significant association between NO concentrations and DAL. Despite the high nitrate content of vegetables, almost 65% of the dietary nitrate (the precursor for NO) could be excreted in the urine, and the remaining 35% might not be converted completely to NO^59^. Therefore, the dietary proportion for NO production is not as much as that of endogenous l-arginine. Another mechanism might be increased levels of cortisol in response to the high acidity of the diet^60^. Cortisol might negatively affect migraine headache recovery^61^. Studies have also suggested that elevated levels of cortisol could be linked to high BMI and blood pressure that have adverse effects on the pathogenesis of migraines^62,63^. Another possible mechanism is the imbalance of gut microbiota, possibly because of lower consumption of fruit and vegetables, in persons with higher DAL. Prior evidence has suggested that gut microbiota are related to migraines^64^. Furthermore, patients with higher DAL were found to have lower magnesium intake, in our study. It has been shown that in those experiencing migraine attacks, brain magnesium levels declined^65^. This is mainly due to the role of magnesium in the human buffering system that acts to balance the acid–base equilibrium under acidic conditions^66^.

There are some strengths and limitations that need to be addressed. First, this is the first investigation that has examined the association between two different measures of DAL and migraine headache frequency, duration, severity, and NO concentrations. Second, dietary intake and the outcomes of interest were assessed through the application of validated questionnaires and laboratory tests. Finally, several possible confounders were taken into account in our analysis. Notwithstanding these strengths, we should acknowledge some limitations in the interpretation of our findings. First, as we have previously mentioned, migraine patients might change their food intake preferences, and thus, the causal relationship cannot be inferred due to the cross-sectional design of the study. Second, despite using validated questionnaires, patients’ responses were subjective, based on their memory, which could lead to inevitable measurement errors. Third, there might still be some residual confounders that we did not consider in our analysis. Finally, although both male and female patients were included in our investigation, we were not able to do a sex-stratified analysis because of the small male sample size. This study was performed on Iranian patients suffering from migraine, therefore, extrapolation of our results to other populations should be made with caution.

## Conclusion

In conclusion, we found PRAL and NEAP to be significantly related to migraine headache frequency. We also observed a significant association between NEAP and HIT-6 scores. However, we did not detect such associations in the case of migraine headache duration and severity, as well as NO concentrations. Further well-designed studies on different populations are needed to confirm our findings.

## Data Availability

The data that support the findings of this study are available from the corresponding author upon reasonable request.
